# Spectroscopic study and molecular simulation: Bovine serum albumin binding with anticancer Pt complex of amyl dithiocarbamate ligand

**DOI:** 10.1016/j.heliyon.2023.e20090

**Published:** 2023-09-13

**Authors:** Zahra Arabpour Shiraz, Nasrin Sohrabi, Mahboube Eslami Moghadam, Mohsen Oftadeh

**Affiliations:** a Chemistry Department, Payame Noor University, 19395-4697, Tehran, Iran; b Chemistry & Chemical Engineering Research Center of Iran, Tehran, Iran

**Keywords:** Bovine serum albumin, Docking simulation, Anticancer Pt compound, Dithiocarbamate

## Abstract

Until now, many methods have been proposed to treat cancer, such as radiation therapy and drug therapy, but none of them have caused a complete cure for cancer. Heavy metal complexes such as cisplatin are among the compounds used as drugs in chemotherapy against cancer cells. These compounds cause cell death and have anti-cancer properties, but they have side effects. The biochemical mechanism of cisplatin is related to its interaction with DNA through covalent binding. To reduce the toxicity of metallodrugs, new complexes can be designed containing S, S- bidentate ligands such as diethyldithiocarbamate. Moreover, anti-cancer compounds probably interact with proteins, such as HSA, before passing the cancerous cell membrane and DNA as a target. So, the function of proteins and their stabilities are expected to change. In this research, the mode of binding of [Pt (bpy) (amyl.dtc)]NO_3_ complex with BSA was evaluated by various thermodynamic methods. Negative binding enthalpy and entropy changes amounts show that the connection between the Platinum compound and BSA occurs via the van Der Waals type of hydrogen bond. The negative Gibbs free energy change was obtained through isothermal titration, which showed interaction proceeds spontaneously. Moreover, the emission titration data showed that protein fluorescence quenching by platinum agent titration is static. Binding, quenching constants, and binding site number were obtained by the Stern-Volmer equation, and only one binding site was determined for this interaction. A Scatchard plot with a positive slope shows the Pt agent-BSA formation is proceeding positively cooperative. The kinetic study displayed that the absorption monitoring followed the second-order model. Finally, molecular docking simulation showed that the position of the Pt agent on protein is placed I under region II.

## 1 Introduction

Cancer, which occurs with the uncontrolled growth and proliferation of cells in a part of the body, results from environmental factors and genetic disorders; today, it is considered the main cause of death in the world [1,2]. Cisplatin is one of the inorganic compounds still sold as an anti-cancer drug; However, in general, this class of drugs is associated with side effects such as gastrointestinal complications and has high treatment costs [3]. Although cisplatin has a good effect on some cancers (such as ovarian and stomach cancer), it has an irreversible destructive effect. It affects the kidneys, and its side effects include toxic effects on kidneys, hair loss, hemolytic anemia, nerve damage, and electrolyte imbalance, and its use is limited due to these toxic effects. Therefore, its performance can be improved by reducing the side effects and increasing the drug level [4,5]. Cisplatin can bond with biomolecules containing S atoms, which inactivates it and reduces toxicity [[6], [7], [8]]. So, many ligand and metal compounds have been synthesized and reported as anti-oxidants, anti-esterases, and anti-cancer agents [[9], [10], [11], [12]]. Therefore, using various metals in anti-cancer agents, like Pd, can lead to fewer side effects and toxicity than platinum compounds. Also, using compounds with sulfur-donating ligands, such as thiosulfate or diethyldithiocarbamate [6,13,14]. One of the most abundant proteins in plasma is albumin, whose role is to regulate blood osmotic pressure, and its interaction with small molecules provides useful information about the properties of the complex. The structure of this protein is spherical, which is responsible for binding and transporting various small ligands in the blood [15]. Bovine serum albumin is one of the proteins that has been studied extensively. BSA has been widely used as a typical protein to peruse the aggregation process of proteins [[16], [17], [18]]. Amyloid nanofibers obtained from bovine serum albumin have been used as one of the most important carriers of medicinal substances in chemotherapy, including cancer, and due to their high binding capacity, they are of great interest for both hydrophilic and hydrophobic drugs. BSA, as a carrier protein, has three homologous (II, I, and III) domains, (L9-L1) Loops, and two related to subdomains A and B [[19], [20], [21]].

**Scheme 1:**
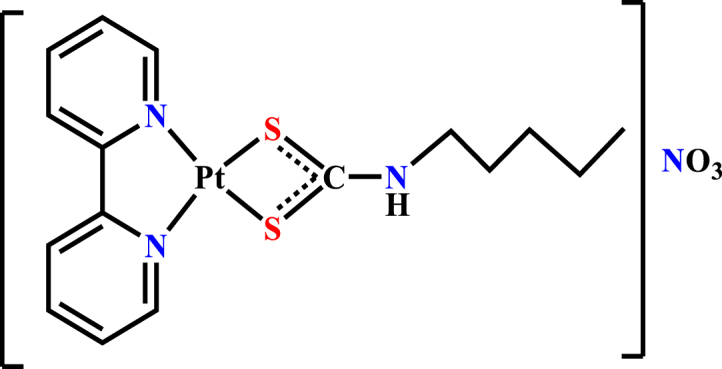
Structure of [Pt(bpy)(amyl.dtc)]NO_3_ complex.

Molecular docking studies are used in drug design and discovery and are important for understanding biological regulatory mechanisms. Since these interactions take place through a specific site on the surface of the macromolecule, to understand their function, we need information about ligand- or complex-protein formation, which can include information such as the number of sets of binding sites and how many binding sites there are in each set. In this study, [Pt(bpy)(amyl.dtc)]NO_3_ (Scheme 1) was selected as an anti-cancer agent, and its interaction with BSA was evaluated by using experimental spectroscopic methods (UV–Vis and fluorescence spectroscopy) based on the report [22], and theoretical docking simulation. The synthesis method and structural data have been presented in Ref. [23]. Cooperativity, the number of binding sites, the Stern-Volmer constant, and thermodynamic and kinetic parameters were evaluated by spectral methods. In addition, the molecule was optimized and analyzed to determine the binding ability and the mode of binding on BSA by a simulation study.

## 2 Experimental section

### 2.1 Material and methods

Recently, [Pt(bpy)(amyl.dtc)]NO_3_ complex has been synthesized and reported as an anti-cancer drug [23]. All materials used in this study were purchased from Sigma-Aldrich (USA). Bovine serum albumin manufactured by Sigma A3782-100 MG was used to prepare BSA solution (1 mg/1 mL Tris buffer). The absorption monitoring was done between 200 and 700 nm by a PerkinElmer Accurately Lambda 25 spectrophotometer (USA). Also, all emissions were detected by a Scinco FS-2 fluorescence spectrophotometer (Korea).

### 2.2 Experimental bovine serum albumin binding

#### 2.2.1 Isothermal titration study

To investigate bovine serum albumin-Pt agent formation, UV absorption was measured in the range of 200–400 nm [22,24]. The protein absorptions were monitored in the absence and presence of various dosages of the Pt complex at 27 and 37 °C.

#### 2.2.2 Thermal stability study

In this step, Tm, the temperature at which half of BSA unfolded, was evaluated [25,26]. The absorption of both Solutions of BSA (2 mg/mL) and BSA-[Pt(bpy)(amyl.dtc)]NO_3_ were detected at 278 nm by the temperature increasing from 25 to 60 °C, separately, where the complex concentration is 1 mM.

#### 2.2.3 Fluorescence spectroscopy

To record the fluorescence emission spectrum of BSA, the range of the fluorescence spectrum was adjusted between 200 and 700 nm (λex = 280 nm), and the width of the excitation and emission gap was set to 5 nm [[27], [28], [29]]. 1500 μl of 0.2 mg/mL BSA solution was placed in the cell, and its emission spectrum was followed by titration of 50 μL Pt agent (10^−6^ M) injection, and the emission spectrum was recorded.

### 2.3 BSA-Pt complex computer simulation

One of the promising technologies in the drug development process is computer-aided drug discovery [[30], [31], [32]]. The advanced AutoDock program is one of the most important molecular docking software at the international level, which is very useful in the discovery and rational development of pharmaceutical molecules. This software can calculate the number and type of chemical interactions between the ligand and the macromolecular receptor (proteins or nucleic acids) based on the binding free energy using force field functions. Before any simulation, the structure of the complex must be optimized. To get the most stable conformations of the Pt complex the structure-optimizing calculations were carried out by applying DFT/B3LYP method with 6–31G and LANL2DZ basis sets [33]. Pt (II) complex was used as a ligand, and BSA as a receptor in the molecular docking. BSA protein with PDB ID: 4F5S was downloaded as a PDB file from the protein database. Interaction of the BSA with Pt (II) complex was performed by the molecular docking program AutoDock 4.2.3 package. To perform molecular docking, the first box building was done with the maximum box size to perform blind docking, and by analyzing the results, the box size and the binding site of the complex to the protein were determined. The main docking and the final analysis were then performed.

## 3 Result and discussion

### 3.1 Binding isotherm of BSA to Pt-complex

**Fig. 1:**
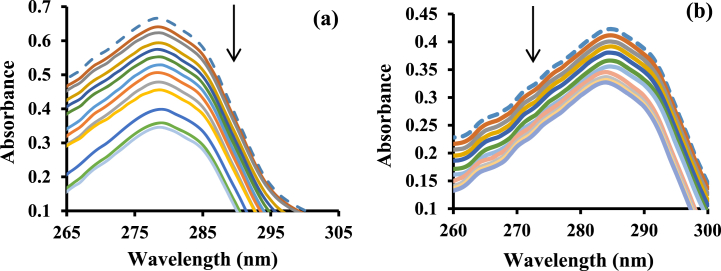
UV–Vis monitoring of BSA (15 μ M) interactions (-- -) by [Pt(bpy)(amyl.dtc)]NO_3_ titrating (4–40 μM) at (a) 27 and (b) 37 °C and in Tris buffer (pH = 7.4). The arrow illustrates the direction of change in intensity upon increasing the concentration of the complex.

Serum albumins are the carriers of numerous drugs. Because of sharp absorption at 280 nm [34], UV/Vis methods make it easy to study to obtain more information on drug-protein interactions. Fig. 1 shows the UV–vis monitoring of BSA in the attendance and absence of various concentrations of the [Pt(bpy)(amyl.dtc)]NO_3_ complex. The decreasing trend in the diagram (Fig. 1a and b) shows that a new complex protein is formed. By using the A_max_ diagram in terms of ʋ،(ʋ = [*complex*]_*b*_/[*BSA*]_*t*_), the values of L_1/2_ (the amount of the complex which can make half of BSA abnormal) were obtained. The less dosage leads to fewer side effects if this complex is used as a medicine in cancer treatment [35]. Different thermodynamic parameters such as the binding constant, K_b_, and the standard binding Gibbs free energy changes, ${\Delta G{}^{\circ}}_{b}$ , can be obtained according to equations (1), (2)) [34]:

(1) $$\left(\frac{1}{A-A_{0}}\right)=\left(\frac{1}{A_{\infty }-A_{0}}\right)+\left(\frac{1}{K_{b}\left[A_{\infty }-A_{0}\right]}\right).\left(\frac{1}{\left[\text{complex}\right]}\right)$$

(2) $${\Delta G{}^{\circ}}_{b}=-\text{RTln}\;K_{b}$$

**Fig. 2:**
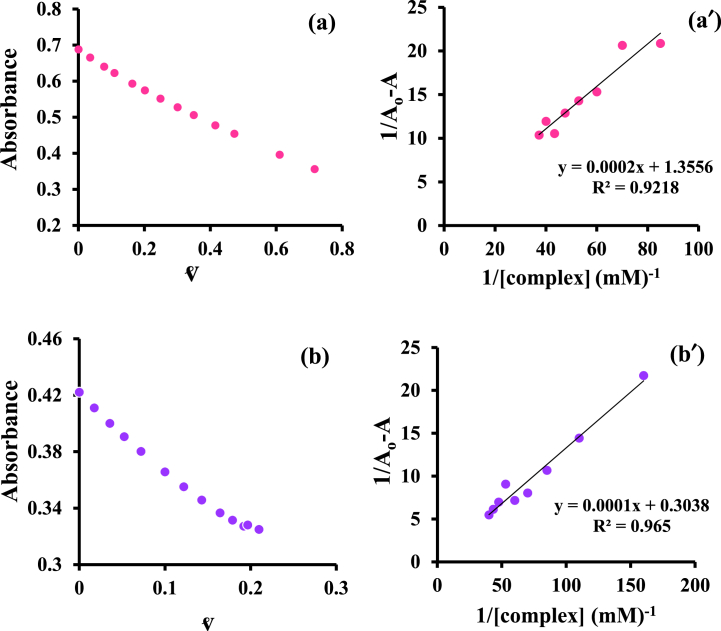
BSA absorbance vs. ʋ and the linear plot of 1/ΔA vs. 1/[complex] at (a, a′) 27 and (b, b′) 37 °C.

**Fig. 3:**
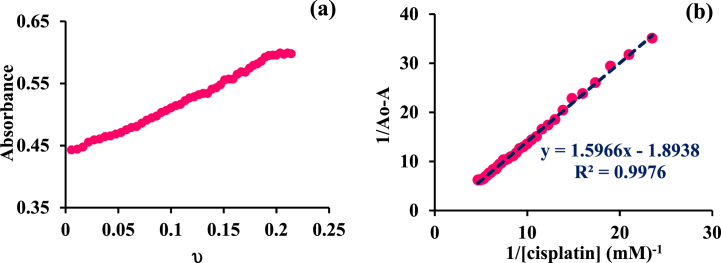
(a) HSA absorbance vs. ʋ at 25 °C and (b) the linear plot of 1/ΔA vs. 1/[cisplatin].

In equation (1), A_0_ is, the initial absorbance of free BSA, A_∞_ is the final absorbance of bound BSA at 280 nm, and A is the measured absorbance at particular complex concentrations (Fig. 2a and b). By drawing the plot of 1/*A*-*A*_0_ vs 1/[complex], Fig. 2a′ and 2b′, the binding constant was obtained. According to these graphs and the binding constant reported in Table 1, it can be concluded rising temperature leads to decreasing binding constant, and the larger binding constant shows a strong interaction between the Pt complex and BSA protein [31]. To compare this data with a clinical Pt drug, cisplatin, this investigation was done with Human Serum Albumin, HSA, at room temperature [35], reported in Fig. 3a and b, respectively. According to Fig. 2, Fig. 3, K_b_ can be obtained. With the help of ΔG^°^_b_ and K_b_ values, other thermodynamic parameters were determined through the van't Hoff condition [36] (equations (3), (4))):

(3) $$\Delta G_{b}^{{}^{\circ}}=\Delta H_{b}^{{}^{\circ}}-T\Delta S_{b}^{{}^{\circ}}=-RTLnK_{b}$$

(4) $$\Delta H^{0}=\left(\Delta {G_{T}^{0}}_{1}/T_{1}-\Delta G_{T2}^{0}/T_{2}\right)/\left(1/T_{1}-1/T_{2}\right)$$

**Table 1:** Experimental half-saturation concentration L_1/2_ and thermodynamic parameters values of BSA-[Pt (bpy) (amyl.dtc)]NO_3_ and HSA-cisplatin formation.

Complex	T (K)	[L]_1/2_	K_b_ M^−1^))	${\Delta G}_{b}^{{}^{\circ}}$ (kJmol^−1^)	${\Delta H}_{b}^{{}^{\circ}}$ (kJmol^−1^)	${\Delta S}_{b}^{{}^{\circ}}$ (Jmol^−1^K^−1^)
[Pt (bpy) (amyl.dtc)]NO_3_	300	0.1	6778	−22	-62.1	-133.6
	310	0.11	3038	−22.7		−126.9
Cisplatin	298	0.18	1183	−17.6	–	–

Thermodynamic parameters are presented in Table 1. According to these values, the type of connection can be determined [36]. The results display that the main forces driving the interaction are van der Waals and hydrogen binding forces [37].

### 3.2 Binding isotherm

The binding analysis of experimental data is done by binding isotherms and Scatchard diagrams, which are always associated with problems. Still, one fact that is very popular today is the concept of binding capacity [38]. The binding capacity can be gotten using equation (5) [38]:

(5) $$\theta =\frac{n_{H}\nu \left(g-\nu \right)}{gRT}$$

Where ʋ is defined by equation (6) [22] as follows:

(6) $$\upsilon =\frac{{\left[\text{ligand}\right]}_{b}}{{\left[\text{protein}\right]}_{t}}$$

**Fig. 4:**
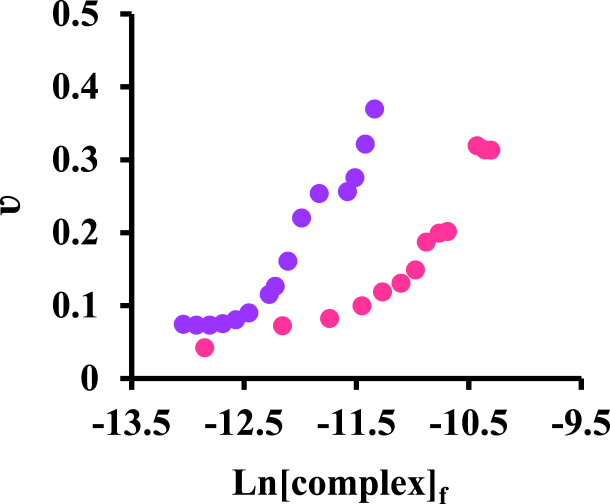
Binding isotherm of BSA with Pt-complex at 37 °C () and 27 °C ().

Therefore, considering the slope of the isotherm plot (Fig. 4), the binding capacity value can be obtained.

Since the binding isotherms drawn at two temperatures are single steps, this system has one binding set [38]. Equation (5) can be rewritten to the linear form (equation (7)) as follow:

(7) $$\frac{RT\theta }{\nu }=n_{H}-\frac{n_{H}\nu }{g}$$

**Fig. 5:**
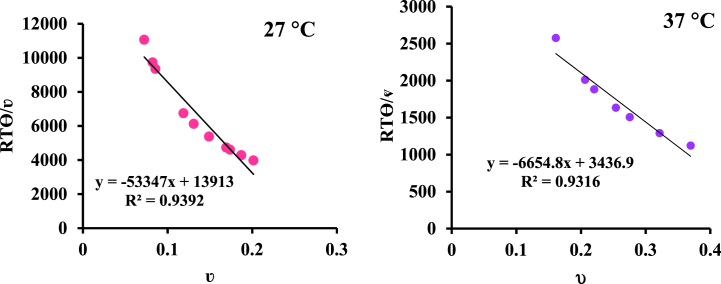
Changes of RTθ/ν in terms of ν for the interaction of BSA with [Pt(bpy)(amyl.dtc)]NO_3_ complex at (a) 27 and (b) 37 °C.

In this expression, n_H_ is called the Hill coefficient. According to this equation, if a system is identical and dependent, the diagram of RTθ/ʋ vs ʋ should be linear (Fig. 5a and b).

Due to the linearity of the binding capacity diagram, RTθ/ʋ in terms of ʋ, only one proper set of binding sites was observed on BSA for this complex [39].

### 3.3 Binding analysis with scatchard diagram

**Fig. 6:**
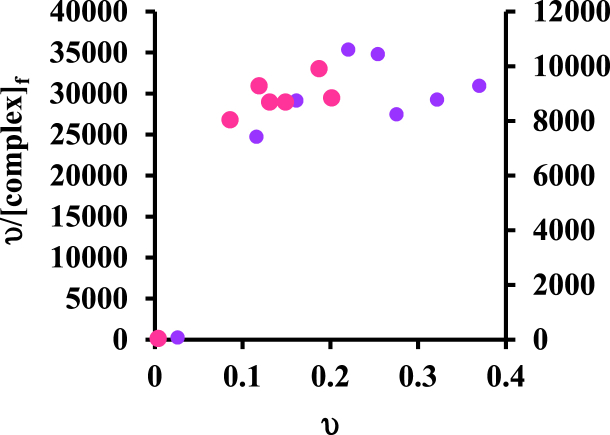
Schematic Scatchard plot of protein binding with of [Pt(bpy)(amyl.dtc)]NO_3_ complex at temperatures 37 °C () and 27 °C ().

By drawing the Scatchard plot (υ/[L] vs υ), we can obtain main data on protein binding with drugs, such as the number of binding sites and the binding constant. So, if this graph is linear, it is non-cooperative; if the slope of the graph is positive and the curve of the graph is downward, it shows positive cooperation, and the opposite case shows negative cooperation. The Scatchard diagram is drawn for the interaction of this complex with bovine serum albumin at two temperatures of 27 and 37 °C (Fig. 6). We use Hill's equation (equation (8)) to obtain Hill binding constant, K_H,_ and Hill coefficient, n_H,_ as follows [6]:

(8) $$\ln\left(\frac{\nu }{g-\nu }\right)=\ln\;K_{H}+n_{H}\;\ln\;{\left[L\right]}_{f}$$

Considering the positive slope of the graph and the low depression of the graphs, it can be concluded that this interaction proceeds via a positive, cooperative mechanism [6,40].

**Fig. 7:**
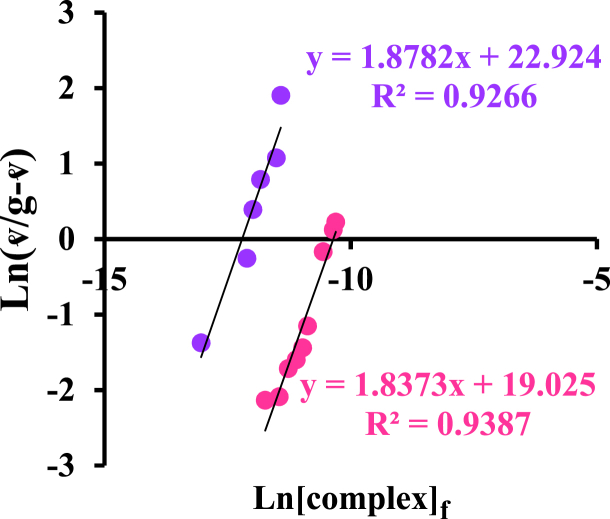
Hill plot of protein binding with [Pt (bpy) (amyl.dtc)]NO_3_ complex at 37 °C () and 27 °C ().

By drawing the graph of Ln (ʋ/g-ʋ) in terms of Ln [complex]_f_ (Fig. 7), Hill's constant, K_H_, and Hill's coefficient, n_H_, were obtained. These values are reported in Table 2. In these graphs, the Hill coefficient is more than one at 27 and 37 °C, with positive, cooperative behavior in this interaction [41].

**Table 2:** Hill parameter and binding constant of BSA interaction with complex.

complex	T (°C)	g	K_b_ (M^−1^)	K_H_ (M^−1^)	n_H_
[Pt(bpy)(amyl.dtc)]NO_3_	27 37	3.8 1.9	6.7 × 10^2^ 3.04 × 10^2^	19.1 22.92	1.8 1.8

The values related to molar and binding Gibbs free energy are obtained through equation (9) as follow [38,42]:

(9) $$\Delta G_{b}^{0}=-RTn_{H}\;\ln\;K_{H}+RT\left(1-n_{H}\right)\ln\;{\left[complex\right]}_{f}$$

**Fig. 8:**
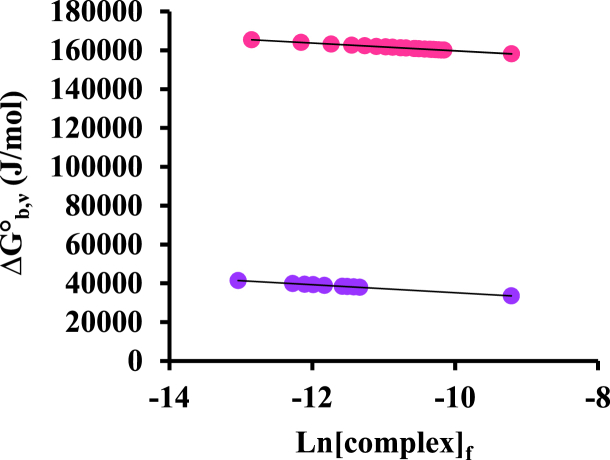
ΔG^°^_b,υ_ graph versus Ln [complex]_f_ for BSA interaction with [Pt (bpy) (amyl.dtc)]NO_3_ at two temperatures of 27 (●) and 37 (●) °C.

Fig. 8 shows the graph of molar binding Gibbs free energy changes versus Ln [complex]_f_ at both mentioned temperatures. The negative slope indicates positive cooperativity in related interactions [43].

### 3.4 BSA thermal stability

**Fig. 9:**
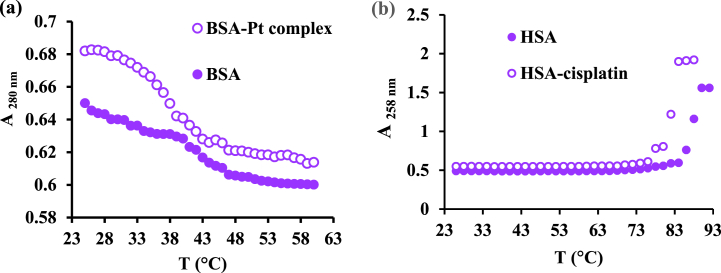
Absorption changes (at 280 nm) vs temperature for (a) BSA in the absence and the presence of [Pt (bpy) (amyl.dtc)]NO_3_ from 25 to 65 °C and (b) HSA-cisplatin from 25 to 95 °C.

The thermal stability of protein can be studied based on [L]_1/2_ value and the ratio of [L]/[BSA] [42] in the absence and presence of the drug. T_m_ was determined by UV scanning of 2 mg/mL solution of BSA at 278 nm in the presence and absence of 10^−4^ M Platinum agent. In this test, the temperature increased from the ambient temperature by 1 °C per minute until it reached 60 °C. The value of Tm for the BSA in the presence of the platinum agent is about 47 °C, while this value is only about 37 °C for the protein (Fig. 9a). This study was repeated using cisplatin as a controller, and HSA thermal changing was continued to 95 °C (Fig. 9b) [35]. The results show Pt complex-BSA formation leads to a stable system [42].

### 3.5 UV–visible kinetic study

**Fig. 10:**
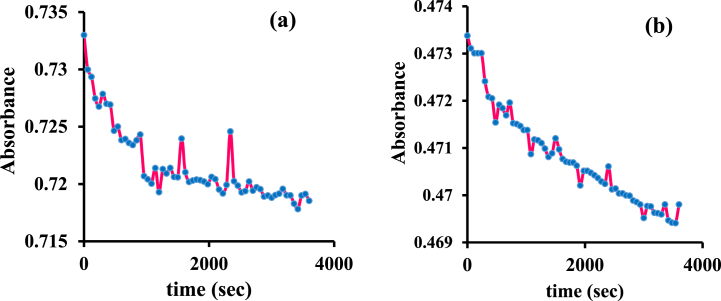
Absorption changes over time in the interaction of [Pt (bpy) (amyl.dtc)]NO_3_ with BSA at (a) 27 and (b) 37 °C.

Kinetic studies consider arranged information about the binding mode of BSA with Pt agent by the time checking recorded 1 min at 278 nm wavelength until 1 h (Fig. 10a and b) [39,43]. Based on equations (10), (11)) and plotting the charts vs time (seconds), the rate constant of the BSA-Pt agent can be determined.

(10) $$\frac{1}{A_{\infty }-A}=\frac{akt}{A_{\infty }-A}+\frac{1}{A_{\infty }-A_{0}}$$

(11) $$\mathrm{Ln}\left(A_{\infty }-A\right)=-akt+\mathrm{Ln}\left(A_{\infty }-A_{0}\right)$$

**Fig. 11:**
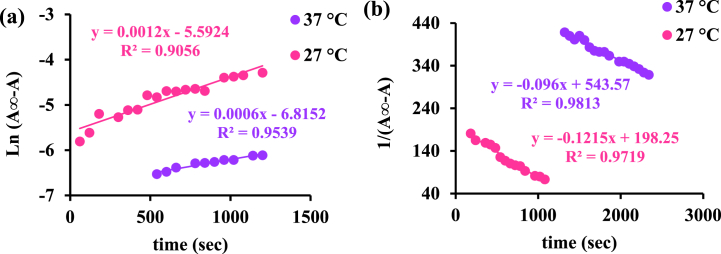
Investigating the first-order (a) and second-order (b) kinetics model in the interaction of [Pt (bpy) (amyl.dtc)]NO_3_ complex with BSA at two temperatures of 27 () and 37 °C ().

In these two formulas, A_0_ is initial absorption, A_∞_ is final absorption, A is absorption in successive times, k is a rate constant, and t is time. To determine the order type of the mechanism in the interaction of the above complex with bovine serum albumin, the following graphs were drawn at two temperatures of 27 and 37 °C (Fig. 11a and b). According to equations (10), (11)), since the square linear regression coefficient, R^2^, in these graphs is more linear for the second-order reaction than the first-order reaction, it is concluded that the kinetics is a second-order type for both 27 and 37 °C during occupying of all binding sites. In biological systems, protein interaction should depend on drug and protein concentrations in binding. So, it will lead to limit protein binding and interaction with drugs. It means probably side effects decrease since this matter is important in research in the drug design field [44].

### 3.6 BSA emission quenching

**Fig. 12:**
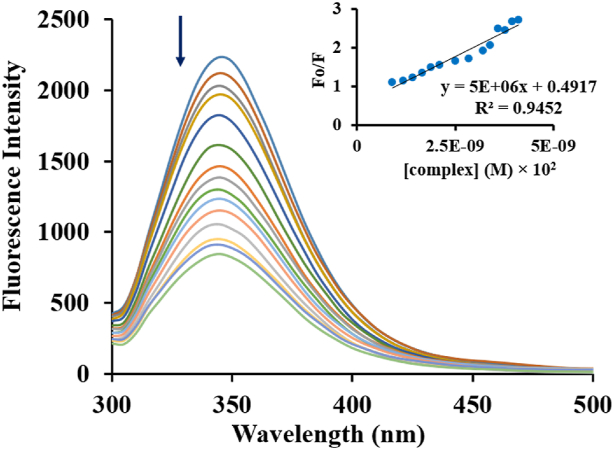
Fluorescence quenching of BSA (λex = 280 nm) in the presence of various concentrations of Pt compound (0–210 μΜ); insert: Stern-Volmer diagrammatic of protein quenching.

Bovine serum albumin has amino acids that have fluorescent properties, so fluorescence spectroscopy is widely used to evaluate its interaction with metal compounds [43]. There are two types of tryptophan in BSA: tryptophan 212, which is in the hydrophobic part, and tryptophan 134, which is on the molecule's surface [44,45]. So, fluorescence emission monitoring was done for BSA-free and BSA-complex systems, and decreasing intensity was observed, along with increasing complex concentration, in the BSA-Pt agent. In this test, we set the excitation wavelength to 280 nm (Fig. 12), and the quenching indicates drug binding to the protein [22].

The Stern-Volmer equation (equation (12)) was utilized to evaluate the experimentally obtained emission data, and the fluorescence quenching proceed [6,40,41]:

(12) $$\frac{F_{0}}{F}=1+K_{sv}=1+k_{q}\tau_{0}\left[Q\right]$$

In this relationship, F_o_, F, τ_0_, Q, k_q_, K_sv,_ and n are fluorescence emissions in the absence and presence of the Pt complex, the lifetime (10^−8^ s) of the fluorophore within the nonappearance of Pt agent, as ligand, which is equal to, the concentration of the ligand, the rate constant; and quenching constant and binding site numbers on protein, respectively [46]. To obtain K_sv_, low concentration regions are considered at the ratio of 1:1 (ligand/protein), and linear F_o_/F plot vs [Q] in various by reducing the fluorescence emission. Based on Ksv = kq.τ equation, kq is also calculated (more than 2 × 10^10^ M^−1^S^−1^), and static interaction is confirmed [47,48].

Using different methods, K_b_ and the number of binding sites per protein molecule can be determined by equation (13), which has been applied as follows:

(13) $$\log\left(\frac{F_{0}-F}{F}\right)=\log\;K_{b}+n\;\log\left[Q\right]$$

In this respect, F_o_ and F, respectively, show the intensity of the fluorescence in the absence and presence of the Ligand, and the [Q] parameters represent the quencher ligand concentration.

**Fig. 13:**
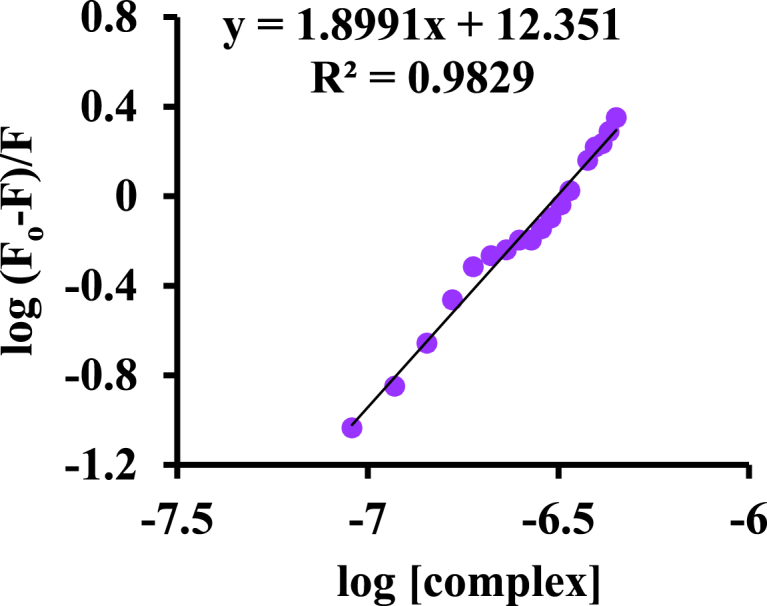
Linear diagram of $\mathrm{Log}\left(\frac{\left(F_{0}-F\right)}{F}\right)$ vs Log [complex].

Based on equation (13), the values of n and K_b_ are evaluated [49] by plotting experimental emission data as the Log $\left(\frac{\left(F_{0}-F\right)}{F}\right)$ vs the log [Q] as shown in Fig. 13 (K_b_ = 5 × 10^6^ M^−1^ and n = 1.3). Also, K_b_ values exhibit the strength of the interaction between BSA and Pt agent [50,51].

### 3.7 Molecular docking study

**Fig. 14:**
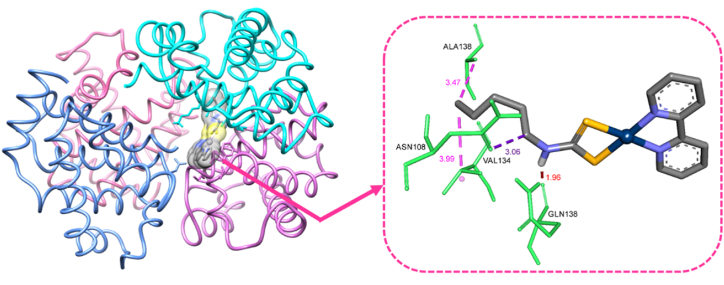
Docking pose of Pt complex interaction with HSA through hydrogen and hydrophobic binding.

The advanced AutoDock program is one of the most important docking simulation software at the international level, which is very useful in the discovery and rational development of pharmaceutical molecules. Using force field functions, this software can calculate the chemical interactions between the ligand and the macromolecular receptor (proteins or nucleic acids) based on the binding free energy [52,53]. The BSA code, which was downloaded, is 4F5S from the PDB. During the data analysis, the appropriate grid size was set with a distance of 0.375 Å along the three axes. Concurring with the Gibbs energy, the restraint consistency was calculated, and the esteem of K_i_ for the Pt agent was 41.29 nM (nanomolar). As shown in Fig. 14, there was no hydrogen bound. In this interaction, one hydrogen bond with the amino acid glutamine and eight hydrophobic bonds with the amino acids alanine, asparagine, glycine, lysine, phenylalanine, and valine were observed. The comes about of molecular docking indicates the binding position of the Pt compound on BSA site I within the subdomain IIA.

## 4 Conclusions

Cisplatin treats human cancers such as bladder and ovarian cancer, head and neck cancer, and testicular cancer. But its clinical utilization is constrained due to a few genuine side impacts. Hence, it is conceivable to utilize S, S giver ligands such as dithiocarbamate to get ready anti-cancer drugs and decrease a few side impacts. In this research, the interaction ability of [Pt (bpy) (amyl.dtc)]NO_3_ with BSA was investigated by using fluorescence emission titration, UV–Vis. absorption monitoring, and molecular docking simulation. Thermodynamic parameters were obtained. The enthalpy and entropy values were −62.1, −133.58 kJ/mol, and −126.91 kJ/mol^.^K at 27 and 37 °C, respectively, which display that the driving forces during the interaction are van der Waals and hydrogen binding. The negative Gibbs free energy obtained showed a spontaneous interaction. Using the spectroscopic kinetic model, the linear regression coefficient in the graph for the second-order reaction is higher than the first-order reaction, so it can be concluded that the kinetic prefers second order, and binding of the complex on BSA depends on drug and protein concentrations. Moreover, the emission titration results showed that protein fluorescence quenching by the Pt complex is a static quenching mechanism by the Stern-Volmer equation. The results showed that there is only one binding site in the interaction of the complex with BSA. Molecular docking showed that the location of this complex on BSA is position I under region IIA.

## Funding statement

The work was supported by 10.13039/501100004220Payame Noor University and the Chemistry and Chemical Engineering Research Center of Iran.

## Authors contributions

*Zahra Arabpour Shiraz:* Performed the experiments, Analyzed the data, and wrote the Experimental sections.

*Nasrin Sohrabi:* Designed the experiment , analyzed all data and interpreted the data and wrote and edited the paper.

*Mahboube Eslami Moghadam:* Prepared materials, analyzed the theoretical data and wrote and edited all the paper.

*Mohsen Oftadeh:* Analyzed tools or data and wrote the titration section.

## Data availability statement

Data will be made available on request.

## Ethics approval

Not applicable. This article does not contain any studies with human participants or animals performed by any of the authors.

## Declaration of competing interest

There is not any conflict in reviewing our manuscripts, such as former advisors, students, or recent collaborators.

## Abbreviations

amyl.dtc: n-pentyl dithiocarbamate
BSA: Bovine Serum Albumin
bpy: bipyridine
dtc: Dithiocarbamate
LGA: Lamarckian genetic algorithm
PDB: Protein Data Bank
Panc1: Human pancreatic cell line
T_m_: melting temperature
UV–vis: ultraviolet–visible
